# Supplementary material for: Transcriptomic Insights into Mechanisms of Early Seed Maturation in the Garden Pea (Pisum sativum L.)
Source: Cells. 2020 Mar 23;9(3):779. doi: 10.3390/cells9030779 (PMC7140803; doi:10.3390/cells9030779)

Zhongwan−6(10)

Global Summary

%DE = 0.82

genes with fdr < 0.2 = 1680 (691 + / 989 −)

genes with fdr < 0.1 = 1567 (628 + / 939 −)

genes with fdr < 0.05 = 1409 (532 + / 877 −)

genes with fdr < 0.01 = 1106 (363 + / 743 −)

genes in genesets = 0

<FC> = 0

<t−score> = −165061.96

<p−value> = 0

<fdr> = 0.18

Global Genelist

Rank	ID	log(FC)	p−value	fdr	Metagene	Description
1	comp22899_c	−0.77	0e+00	2e−13	30 x 1	
2	comp24437_c	−0.1	0e+00	2e−13	12 x 29	
3	comp38522_c	−0.04	0e+00	2e−13	22 x 15	
4	comp40422_c	−0.36	0e+00	2e−13	30 x 25	
5	comp45569_c	−0.11	0e+00	2e−13	7 x 29	
6	TRINITY_DN1	−1.54	4e−16	6e−13	30 x 30	
7	TRINITY_DN7	−0.95	5e−15	6e−13	30 x 1	
8	TRINITY_DN6	−0.84	6e−15	6e−13	30 x 30	
9	comp19514_c	−0.04	7e−15	6e−13	23 x 18	
10	comp39208_c	−0.03	7e−15	6e−13	23 x 14	
11	TRINITY_DN2	−0.31	8e−15	3e−12	30 x 16	
12	TRINITY_DN8	−0.69	2e−14	3e−12	30 x 1	
13	TRINITY_DN1	−0.37	2e−14	3e−12	30 x 25	
14	TRINITY_DN1	−0.09	4e−14	3e−12	7 x 30	
15	TRINITY_DN1	−0.25	4e−14	3e−12	30 x 6	
16	TRINITY_DN1	−0.19	4e−14	3e−12	29 x 15	
17	TRINITY_DN1	−0.17	5e−14	4e−12	26 x 28	
18	comp20491_c	−0.01	6e−14	1e−11	15 x 22	
19	TRINITY_DN9	−0.07	1e−13	1e−11	28 x 13	
20	TRINITY_DN3	−0.09	2e−13	1e−11	30 x 16	

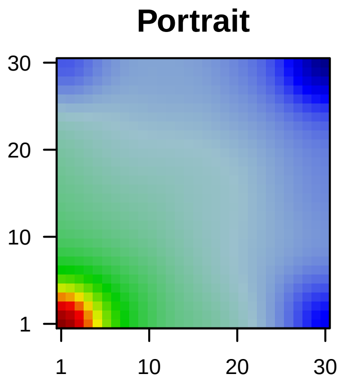

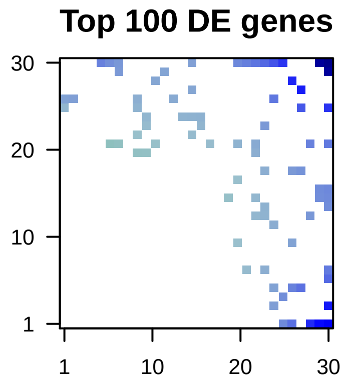

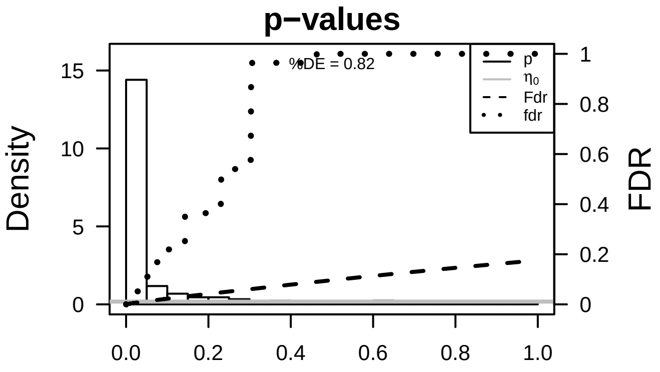

Supplement: Supplementary file 1 [file cells-09-00779-s001.zip › Supplementary materials/FileS4.zip!/FileS3/TFs - Results/Summary Sheets - Groups/Reports/Zhongwan.6.10..pdf]
